# How women respond to uncertainty in the context of genetic screening: A qualitative analysis framed by the uncertainty tolerance model

**DOI:** 10.1002/jgc4.70110

**Published:** 2025-09-08

**Authors:** Yi Liao, Anne C. Madeo, Lingzi Zhong, Wendy Kohlmann, Erin Rothwell, Kimberly A. Kaphingst

**Affiliations:** ^1^ Department of Communication University of Utah Salt Lake City Utah USA; ^2^ Department of Population Health Sciences University of Utah Salt Lake City Utah USA; ^3^ Department of Communication University of Minnesota Duluth Duluth Minnesota USA; ^4^ Clinical Cancer Genetics Service Veterans TeleOncology Program Durham North Carolina USA; ^5^ Cancer Control & Population Sciences Huntsman Cancer Institute Salt Lake City Utah USA; ^6^ Department of Obstetrics and Gynecology University of Utah Salt Lake City Utah USA

**Keywords:** cancer prevention, genetic screening, reproduction, uncertainty, uncertainty tolerance

## Abstract

With advances in next‐generation sequencing technologies, individuals can seek genetic risk information for multiple conditions. However, feasibility and communication challenges could arise if offering multiple genetic tests simultaneously, such as cancer predisposition testing and carrier screening for pregnancy planning. Genetic screening introduces uncertainty from probabilistic results, ambiguous gene‐disease associations, and complex variant interpretation, intertwining with psychosocial concerns impacting decision‐making and emotional well‐being. This study utilized coding reliability thematic analysis with both a deductive and inductive approach using the uncertainty tolerance model as a framework to explore how reproductive‐age women perceive and respond to uncertainty in the context of genetic screening. Through in‐depth interviews with 20 women recruited from obstetrics/gynecology clinics, the study revealed cognitive, emotional, and behavioral responses to uncertainty. Participants lacked familiarity with genetic screening but expressed interest in learning more. Positive cognitive responses were associated with desires for proactive health management, while negative responses often stemmed from concerns about test accuracy and potential side effects. Emotional responses ranged from hope and excitement to fear and anxiety, shaping information‐seeking behaviors. The study underscores the importance of tailored patient education and communication strategies in genetic counseling to address uncertainty, support informed choice, and alleviate distress. The findings offer insights for improving genetic counseling practices and enhancing patient‐centered care.

## 1 INTRODUCTION

Next‐generation sequencing technologies can provide individuals with risk information for numerous genetic conditions (Lindor et al., 2017). Patients have expressed interest in receiving genetic information in addition to cancer risk (Delanne et al., 2019; Kaphingst et al., 2016). However, genetic counseling research and clinical practice have typically focused on offering testing for a single indication at a time. Although returning multiple genetic test results may increase the perceived value of genetic testing (Bartley et al., 2020), it could also create substantial communication challenges due to greater information volume and complexity (Kaphingst et al., 2016). Consequently, there is a pressing need for further investigation into the uptake of simultaneous multiple genetic tests and the associated genetic counseling outcomes. Such research should encompass various factors, including the perceived utility of services and results, emotional responses, and comprehension when delivering results for multiple indications.

People who are pregnant or planning pregnancy may be particularly interested in various types of genetic information and receiving multiple genetic screenings simultaneously, like cancer predisposition testing (CPT) and carrier screening (CS). CPT provides information about an individual's cancer risks to inform cancer prevention and screening strategies (Rahman, 2014), while CS identifies couples at risk of conceiving a child with serious health conditions (Bajaj & Gross, 2014), which can inform family planning. The two tests share pretest genetic counseling content elements and could be offered in an integrated manner, expanding the potential reach of cancer genetic evaluation in this population.

While genetic screenings provide extensive information and hold promise for early intervention and preventive measures, they also introduce uncertainty into individuals' lives. Uncertainty in genetic testing stems from various sources, including the probabilistic nature of test results, scientific ambiguity surrounding gene‐disease associations, and the complexity of interpreting genetic data (Han et al., 2011; Hodgson et al., 2016; Kaufman et al., 2023; Medendorp et al., 2018). This uncertainty intertwines with psychosocial and existential concerns, impacting individuals' decision‐making processes and emotional well‐being (Bottorff et al., 1998; Braithwaite et al., 2002; Rauscher, 2017; Zhong et al., 2021). For women of reproductive age, considering genetic screening adds layers of complexity as they weigh the potential implications for their health and that of future generations (Hong, 2020; Van Steijvoort et al., 2022).

### 1.1 Genetic screening and uncertainty

Genetic screening is currently offered to individuals with a family history of certain diseases or conditions, as well as for reproductive CS to identify carriers of recessive genetic conditions, even in the absence of family history (Evans et al., 2001; Gregg, 2018; Gregg et al., 2021). The purpose of genetic screening is to identify those at risk of developing a disease themselves or having offspring affected by a genetic condition (Beauchamp et al., 2018; Eng et al., 2001). Specifically, CPT may allow tailored cancer prevention and screening strategies. Early identification of an increased risk for an inherited condition allows for targeted screening, monitoring, and preventive interventions, ultimately reducing morbidity and mortality associated with these conditions (Evans et al., 2001; Medendorp et al., 2018). CS primarily enhances reproductive autonomy by providing individuals and couples with information about their risk of having children with inherited conditions, thereby enabling informed reproductive decision‐making (Henneman et al., 2016; Kraft et al., 2019).

Genetic screening offers individuals valuable insights for making informed decisions, yet it also inevitably introduces uncertainty. For example, genetic screening may provide information about gene variations associated with diseases that lack treatment or prevention, or gene variations whose effects are unclear. Uncertainty refers to a lack of knowledge or information about a situation, event, or outcome, leading to unpredictability or ambiguity in decision‐making (Rowe, 1994). Uncertainty in genetic testing arises from various sources including probability, ambiguity, complexity, and unavailability (Han et al., 2011; Zhong et al., 2020). Examples of this uncertainty include the provision of likelihoods rather than definitive diagnoses in test reports, scientific ambiguity surrounding gene‐disease associations, the complexity of genetic results often necessitating genetic counselors' assistance to interpret, and the absence of prevention and treatment options for certain diseases (Hodgson et al., 2016; Kaufman et al., 2023; Medendorp et al., 2018). Beyond the source of uncertainty, uncertainty in genetic testing intertwines with psychosocial and existential concerns for individuals, encompassing the impact of the illness or treatment on one's life goals, personal relationships, and overall sense of meaning in life (Han et al., 2011).

The uncertainty surrounding genetic screening arises as patients weigh the potential benefits and limitations of undergoing the test, as well as how the results may impact their lives (Hong, 2020). One prominent consequence of this uncertainty is the experience of negative emotions such as fear, anxiety, stress, and depression (Bottorff et al., 1998; Braithwaite et al., 2002; Rauscher, 2017; Zhong et al., 2021). Research has shown that avoiding the feeling of uncertainty plays a significant role in individuals' decisions regarding genetic screening (Wolff et al., 2011). Therefore, it is imperative to investigate what factors contribute to uncertainty and how women of reproductive age respond to it when contemplating genetic testing. Given that patients often rely on healthcare providers to manage uncertainty (Zhong et al., 2020), understanding these questions can offer valuable insights for healthcare providers when communicating regarding genetic screening options and test results.

### 1.2 Uncertainty tolerance and the uncertainty tolerance model

Extensive research has been conducted on uncertainty tolerance (UT) and its relationship to health decision‐making and outcomes (Strout et al., 2018). While some scholars view UT as a personal trait, associating higher UT levels with lower levels of negative emotion and increased intentions for screening tests (Brown & Fernie, 2015; Eisenberg et al., 2015; Han et al., 2014), the relationship is not consistently linear across all contexts. For instance, among men and women of reproductive age, UT has been positively linked to CS uptake (Tambor et al., 1994). However, a more recent study on communicating uncertainty suggests that an individual's UT level does not necessarily moderate the relationship between uncertainty perception and behavioral intention (Han et al., 2018).

Alternatively, Hillen et al. (2017, p. 70) define UT as “the set of negative and positive psychological responses—cognitive, emotional, and behavioral—provoked by the conscious awareness of ignorance about particular aspects of the world.” This definition portrays UT as a multidimensional construct encompassing not only the perception of uncertainty but also the responses to it (Hillen et al., 2017).

**FIGURE 1:**
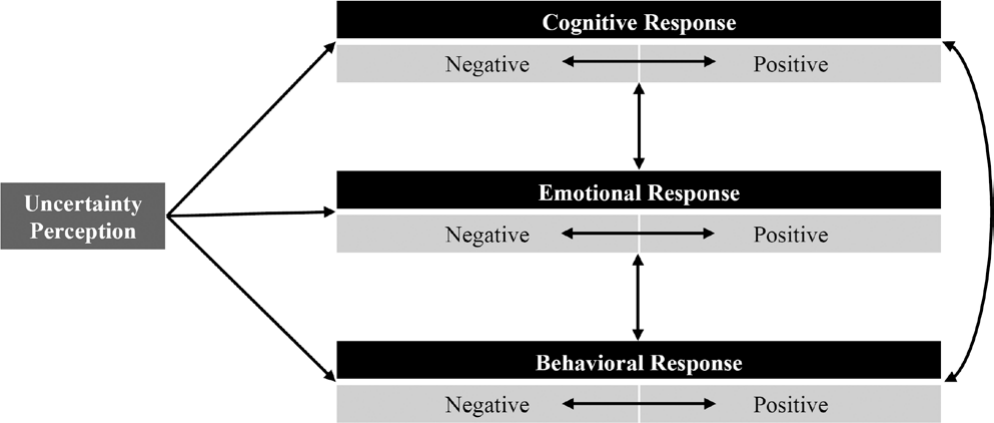
The uncertainty tolerance model (Hillen et al., 2017).

Derived from the definition, the same research team proposed the uncertainty tolerance model (UTM). The model (Figure 1) allows for UT to be approached as either a personality trait or a state contingent on context and situation. Trait‐focused research aims to understand individual differences in UT and tailor interventions accordingly, while state‐focused research investigates contextual influences on UT and develops corresponding interventions. The model accommodates responses occurring at any point in the sequence of events and emphasizes the flexibility of conceptual boundaries around UT.

In the model, uncertainty serves as the overarching and fundamental metacognition concerning a particular object. Following the perception of uncertainty, individuals exhibit cognitive, emotional, and behavioral responses. These responses may occur simultaneously. A positive cognitive response involves exhibiting a favorable attitude toward receiving additional information about genetic screening and intending to undergo genetic screening in the near future. This positive response may manifest as acknowledging the value of genetic screening, viewing it as an opportunity to enhance understanding of disease risk, feeling confident in pursuing genetic testing, or expressing confidence in the actions they decide to take based on the results. Conversely, negative cognitive responses may involve questioning the value of genetic screening, expressing concerns about the procedures and potential side effects of tests, or experiencing a lack of self‐efficacy. Emotional responses encompass a range of states, including positive emotions such as hope and excitement, as well as negative emotions such as worry, fear, and anxiety. Behavioral responses may include positive actions such as seeking information and making decisions, or negative actions such as avoiding information or decision‐making. These responses can be categorized as source‐focused (aimed at altering the uncertain situation) or consequence‐focused (aimed at mitigating the effects). Responses may occur consciously or unconsciously, and individuals may vary in their awareness of how uncertainty influences their thoughts, feelings, and behaviors.

UTM offers a comprehensive framework to assess the full temporal and spatial spectrum of uncertainty as it manifests across various contexts (Hillen et al., 2017). As noted by Hillen et al. (2017), the UTM serves as a conceptual model, but further studies are necessary to elucidate the relationship between the perception of uncertainty and different appraisals, as well as the subsequent cognitive, emotional, or behavioral responses. Under the guidance of the UTM, the current study seeks to investigate the responses of women of reproductive age to uncertainty perception within the context of genetic screening.

### 1.3 Study aims

Drawing upon the UTM, this qualitative study explores the perceptions and responses of women of reproductive age to uncertainty in genetic testing, focusing on cognitive, emotional, and behavioral aspects. In addition to examining cognitive and emotional responses to uncertainty and their influence on information‐seeking behaviors, this study also explores the factors shaping individuals' responses to uncertainty. Examining both CPT and CS within the same study allows us to understand how individuals navigate different categories of genetic risk information and whether decision‐making patterns are consistent across genetic testing domains with varying personal and temporal relevance.

## 2 METHODS

### 2.1 Participant recruitment

After receiving Institutional Review Board approval, participants were recruited from obstetrics and gynecology (OB/GYN) clinics within a university‐affiliated health system. Electronic health records were screened to identify eligible individuals, and eligibility was confirmed before enrollment. Eligible participants were patients of the OB/GYN clinic aged 20–35 years on the screening date, had never been diagnosed with cancer (except nonmelanoma skin cancer), and had not previously received genetic services for CPT or CS. A stratified sampling approach was employed, recruiting a mix of currently pregnant individuals and those with a prior pregnancy, as well as individuals with and without a family history of cancer. We collected both sex assigned at birth and current gender identity during our eligibility screening process. All participants were assigned female at birth and identified as women. We collected the rest of the demographics at the end of each interview.

Participant recruitment ceased after 20 women participated in the interviews, as data sufficiency was achieved (LaDonna et al., 2021), providing both adequate depth in understanding key themes and breadth to identify potential discrepant experiences. All participants completed the interview process with no dropouts. Field notes were made during and after each interview.

### 2.2 Data collection

A non‐Latina White individual who identified as a woman was trained in conducting in‐depth interviews and completed all interviews via Zoom. The interviews took place between June 13 and July 20, 2022, lasting from 14 to 39 min. All participants provided informed consent prior to the interview. The interviews were audio‐recorded, and the recordings were transcribed verbatim.

The interviews followed a semi‐structured format, with the interview guide (see Appendix A) developed by an interdisciplinary research team with expertise in health communication, bioethics, and genetic counseling. The guide was designed to explore participants' interest in and understanding of genetic testing, their decision‐making processes when considering genetic information with varying levels of uncertainty, personal meaning‐making around genetic risk, information and communication preferences, and views on the acceptability and delivery of routine genetic screening. The first set of questions explored participants' attitudes toward receiving information about gene variations with and without treatment or prevention options, as well as those with unknown effects. The subsequent set of questions concentrated on CPT, while the third set focused on CS. Brief introductions about CPT and CS were provided during the interview to ensure all participants received consistent information before responding to questions about each testing type. Practicing genetic counselors reviewed the interview guide. Participants were instructed that for the purposes of the interviews, they should assume insurance would cover the costs of any discussed genetic testing, with the participant paying less than $100, up to a maximum of $250. After their interview, participants received a $25 gift card.

### 2.3 Data analysis

Dedoose, a software for qualitative analysis, was used to facilitate coding reliability thematic analysis, employing a hybrid deductive and inductive approach. This approach is primarily characterized by a structured approach to coding centered around a codebook and a focus on measuring intercoder agreement (Braun & Clarke, 2021). Author 1 drafted an initial codebook based on the UTM and three interview transcripts, which represent a deductive or theory‐driven orientation to coding (Braun & Clarke, 2021). Author 1, Author 2, and Author 3 then met regularly to refine the codebook (see Appendix B) using four additional transcripts. This iterative refinement process introduces an inductive or data‐driven element, allowing for the evolution of codes and a deeper understanding of the data (Braun & Clarke, 2021; Roller, 2019). The final version of the codebook includes seven codes: uncertainty, positive cognitive response, negative cognitive response, positive emotional response, negative emotional response, positive behavioral response, and negative behavioral response. Author 1 (an Asian female with a research focus on health communication) and Author 2 (a White female with over 20 years of experience in genetic counseling) independently coded all the transcripts following a hybrid deductive and inductive approach. The intercoder reliabilities (Cohen's Kappa) range from 0.70 to 0.88 for the seven codes. According to established interpretation guidelines (Sim & Wright, 2005), Kappa values of 0.61–0.80 indicate substantial agreement, while values of 0.81–1.00 represent almost perfect agreement. Our range of 0.70 to 0.88 demonstrates substantial to almost perfect agreement between coders. The analysis also identified themes based on frequently reported codes, how codes co‐occurred, and the relationships among the codes.

## 3 RESULTS

Among the 20 participants, 12 identified themselves as White, while four identified as Hispanic/Latina/Latinx. Fourteen participants had completed a college degree or higher level of education. At the time of the interviews, nine participants were pregnant, and 11 had biological children. Sixteen participants disclosed a family history of cancer, three of whom had a first‐degree relative (e.g., parent or sibling) with such a history. Table 1 provides more details about participant characteristics.

**TABLE 1:** Participant characteristics (*N* = 20).

Race and ethnicity^a^	White (13, 65%)
	Hispanic (4, 20%)
	Asian (3, 15%)
	Pacific Islander (2, 10%)
Education level	Some college or less (6, 30%)
	College degree or higher (14, 70%)
Had biological children	Yes (11, 55%)
	No (9, 45%)
Family cancer history	Yes (16, 80%)
	No (4, 20%)
In pregnancy	Yes (9, 45%)
	No (11, 55%)

^a^ Categories are not mutually exclusive.

**FIGURE 2:**
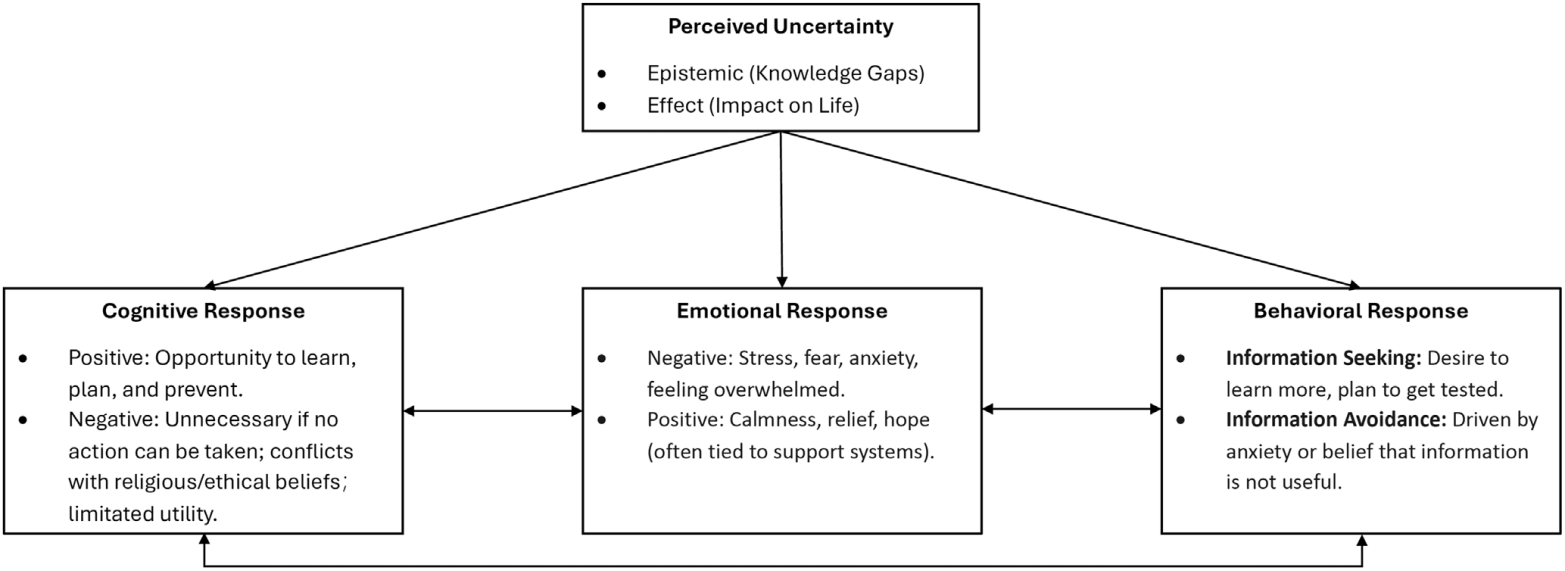
Uncertainty processing in genetic screening.

Data analysis was conducted in March 2024, following the framework of the UTM. The qualitative analysis identified four themes: (1) the primary sources of uncertainty perceived by women of reproductive age when considering genetic testing, (2) participants' cognitive responses toward uncertainty and their interaction with other components in the model, (3) participants' emotional responses toward uncertainty and their interaction with other components in the model, and (4) participants' behavioral responses toward uncertainty and their interaction with other components in the model. Figure 2 provides a visual representation of the dynamic interplay among perceived uncertainty, cognitive, emotional, and behavioral responses.

### 3.1 Theme 1: The primary sources of uncertainty perceived by women of reproductive age when considering genetic testing

Analysis revealed two primary dimensions of uncertainty that shaped participants' perceptions and decision‐making processes regarding genetic screening. Table 2 presents an overview of these uncertainty types, associated themes, and exemplar quotations.

**TABLE 2:** Summary of uncertainty type.

Type	Key themes	Representative quotes
Epistemic uncertainty	Unfamiliarity with procedures Limited gene‐disease knowledge Test reliability	“I don't know much about it [genetic testing], so I don't know” (P19) “[I] don't know which cancers all have been linked genetically” (P2) “I don't know, does that have like a false‐positive kind of thing and how accurate are the‐‐ I don't know” (P15)
Effect uncertainty	Impact on life	“I guess it wouldn't really make a difference for me” (P6) “I don't know, something might happen…” (P1) “it [test result] may impact like some of my health behaviors” (P19)

The majority of participants demonstrated personal epistemic uncertainty with genetic screening procedures, gene‐disease associations, and testing eligibility criteria. Additionally, some of them also demonstrated concerns regarding the reliability, accuracy, and methodological aspects of genetic testing. These concerns encompassed questions about false‐positive rates, testing accuracy, and potential adverse effects. It should be noted that this type of uncertainty could arise when women of reproductive age lack genetic knowledge as well as when they perceive receiving too much genetic information that was inactionable.

Beyond epistemic uncertainty, participants expressed uncertainty regarding how genetic screening results might impact their personal lives and decision‐making processes. This dimension (effect uncertainty) was characterized by participants' inability to predict or envision potential outcomes, as exemplified by one participant's statement: “I don't know, something might happen” (Participant 1). This uncertainty extended to concerns about how results might influence reproductive planning, medical management, and family dynamics.

### 3.2 Theme 2: Participants' cognitive responses toward uncertainty and their interaction with other components in the model

Perceived uncertainty greatly influenced participants' cognitive responses, often reflected in their frequent use of phrases like “I don't know” when evaluating their interest in different types of genetic screening. Despite the uncertainty, most participants demonstrated an interest in learning more about genetic screening, particularly concerning cancer risk and results related to children's health. They perceived genetic screening as an opportunity to assess their health status and potentially prevent adverse outcomes. For example, one woman described the importance of genetic screening, stating,

I think it's just like kind of an annual check for your body, and it's good to know that early rather than later. Maybe at an early stage, we can prolong the process of a healthy life, and we can take action if there is any treatment or prevention method available. It's good to do that early rather than later, I feel. (Participant 11)

Most participants viewed undergoing multiple genetic screenings simultaneously in a positive light, considering it convenient and efficient. As one participant stated, it was a “win–win situation” (Participant 13), as having multiple tests at the same time helped save time by reducing clinic visits. For participants with a fear of blood draws, undergoing multiple screenings at once was also advantageous, as it minimized the number of times they needed to endure that unpleasant experience.

Given the context of the interviews occurring around the release of the Dobbs vs. Jackson Women's Health Organization decision overturning Roe v. Wade, it is noteworthy that most participants completed their interviews within a month of this event. Within this period, participants held diverse views about CS, especially considering that one possible option based on genetic screening results is abortion. One woman believed that having CS before pregnancy would empower individuals to make informed reproductive plans. Conversely, another woman argued that if the state could not provide abortion services, obtaining CS would be unnecessary.

Additionally, participants with religious beliefs also expressed negative cognitive responses toward CS. One participant explained,

I felt like it didn't matter to me to know, that it wouldn't change the way I approach the pregnancy. It wouldn't change the way I prepared for having the baby, and if there was something wrong, once we had the baby, then we want the baby and we will prepare then for it all. So, that was kind of my approach, again, kind of getting back to maybe the religious aspect for me personally. We are financially stable enough, we feel like we are, we have enough family support. Like, we have everything that we would be okay trying to take care of the baby's struggles regardless. And I think from an ethical point of view, it's not our job to decide if someone, like what someone's struggles are. And so, that's why I wouldn't want to do it for my baby. (Participant 3)

#### 3.2.1 Gene variations

Facing gene variations that increase the risk of diseases or health conditions without available treatments, and variations where the effects on health remain unknown, half of the participants remained interested in knowing their genetic predispositions. They held onto the belief that scientists will eventually uncover links or treatments in the future, and they found solace in mentally preparing themselves for potential outcomes.

The other half of the participants exhibited a negative cognitive response. They believed that the results would not benefit them, especially if there is no actionable plan available. As one participant expressed, “But that's not something I feel like I'd care as much about unless there was like something more immediate that I could actually do to help, you know, be the most prepared, or take actions in order to kind of help” (Participant 19). Another significant reason for the lack of interest among participants in variations associated with diseases without treatment and variations with unknown effects is the negative emotions the uncertainty elicits, which will be discussed in the following section.

#### 3.2.2 Family history

Participants unfamiliar with their family history or unable to access that information expressed positive attitudes toward genetic screening. For example, one woman stated,

I'm not too worried based on what I know about our families, but there's stuff in our family genetics we don't know as much about…that's why…some of the family genes come from people we don't know about. So, that would be good to just have awareness. (Participant 8)

Among participants familiar with their family health history, attitudes diverged. Some felt genetic screening was unnecessary since they had little concern about their health status or passing diseases to children. In addition, they were aware of preventive behaviors, and would receive preventive screening. Others believed genetic screening could reveal additional information about their health that they did not already know.

### 3.3 Theme 3: Participants' emotional responses toward uncertainty and their interaction with other components in the model

Facing uncertainty, every participant conveyed negative emotions, using words like “stressful,” “fear,” “scared,” “anxious,” “overwhelmed,” and “worried.” For example, one woman (Participant 17) indicated that the unknown future made her feel stressed about something unexpected happening. The situation was exacerbated for participants with certain self‐described personality traits. As one participant mentioned, “I feel like with me personally‐‐ you know, like I keep saying I worry a lot” (Participant 19). Some participants expressed that during pregnancy, they were “worried about everything” (Participant 12), and the uncertainty added “another layer of stress” (Participant 19).

Participants reported positive emotional reactions less frequently than negative. Some participants expressed calmness regarding what might happen to themselves or their children. This emotional response seemed related to personality or religious beliefs, as expressed by Participant 17, who said, “that's what it was—that's how it was meant to be. Like that's how I guess God wanted it.” Additionally, some participants reported feeling “lucky,” “relieved,” and “hopeful.” These positive emotional responses stemmed from factors such as familiarity with their family health history, the absence of a family history of cancer, having sufficient support from family members, and being in a stable financial situation, which enabled them to cope with the potential occurrence of disease in their offspring.

Some emotions were reactions to cognitive responses. Generally, a positive cognitive response tends to coincide with positive emotions, while a negative cognitive response tends to lead to negative emotions. Some participants believed that acquiring more information could help them prepare, which “puts a lot of ease” (Participant 17) on them and “gives a lot of relief” (Participant 5). On the negative side, knowing the information could lead to “thinking negatively about it” (Participant 3), especially when there are gene variations related to the disease for which there are no prevention methods or when the effects of these variations are unknown. However, sometimes positive cognitive responses and negative emotions, or negative cognitive responses and positive emotions, would occur simultaneously, as participants evaluated the pros and cons of receiving tests. For example, a woman stated, “I would be a little less interested just because of my anxiety. But I might still do it just so that I know what signs to look for in my kids” (Participant 2).

### 3.4 Theme 4: Participants' behavioral responses toward uncertainty and their interaction with other components in the model

Most participants expressed willingness to learn more about their genetic information and screening options. This decision was often directly influenced by feelings of uncertainty about their cancer risk or genetic screening knowledge, as one woman stated: “I don't know much about other genetic tests. So, I would want more information so I could get as many tests as makes sense at the same time” (Participant 8). Some preferred learning at a personal level, viewing exploration of their genetic information as an opportunity for self‐growth and understanding. Additionally, their cognitive response to uncertainty drove their desire to seek more information. Since most participants had positive cognitive responses to genetic screening—seeing it as an opportunity to assess health status, potentially prevent adverse outcomes, and make reproductive plans—they wanted more information, and over half planned to undergo screening within a year if it were offered. Most participants felt that acquiring additional knowledge could alleviate negative emotions, exemplified by: “I think it (genetic screening) puts a little bit more ease on me as a parent, so I wouldn't be surprised if it (disease) happened. If I knew I was a genetic carrier and stuff like that” (Participant 7).

Conversely, information avoidance could result from uncertainty and negative cognitive responses. This commonly occurred when information was related to diseases without treatment, unpreventable and serious conditions like “Huntington's disease” (Participant 2) or “pancreatic cancer” (Participant 12), or gene variations with unknown effects. For CS, the avoidance was sometimes related to pro‐life beliefs. Negative emotions were a major factor driving information avoidance, with some believing more knowledge would make them “too anxious.” During pregnancy, one woman described avoiding testing due to emotional vulnerability. Positive emotions could also prompt avoidance. One participant said, “We just weren't that concerned, so we didn't do genetic testing” (Participant 8).

#### 3.4.1 Attitude toward receiving reading materials before appointments

As a positive behavioral response, receiving educational materials prior to medical appointments can serve as a strategy for coping with and reducing uncertainty surrounding genetic screening and testing. Most participants exhibited a positive cognitive response toward receiving information about genetic screening for hereditary cancer syndromes or carrier status prior to their doctor appointments, either through a website or application. By doing so, they believed they could acquire a basic understanding and “do their own research” (Participant 4) and acquire a basic knowledge before the appointment, enabling them to ask additional questions during the appointment and make more informed decisions. For instance, one woman said, “I think that would be a good idea just like so that when you're at the appointment, you can like know what you want to do and be informed and then just go ahead with your decision right then” (Participant 6).

Some participants offered suggestions regarding accessibility and readability. Participant 10 expressed a preference for flyers with bullet points over applications or websites. Similarly, Participant 18 shared a similar sentiment, mentioning her dislike for installing an application on her phone and preferring “something easy, accessible.” Such preferences may arise from a desire to minimize complexity or ambiguity that could exacerbate feelings of uncertainty.

## 4 DISCUSSION

The current study offers valuable insights into how women of reproductive age perceive and respond to uncertainty in the context of genetic screening, particularly focusing on cognitive, emotional, and behavioral aspects. Drawing upon the UTM, the study explores various dimensions of uncertainty and sheds light on the factors influencing women's attitudes and decision‐making processes regarding genetic testing.

The primary sources of uncertainty identified by participants were perceived epistemic uncertainty, manifesting as unfamiliarity with genetic screening procedures and limited understanding of gene‐disease associations, or inherent limitations or unknowns in current scientific knowledge about genetic screening techniques. These sources align with previous research highlighting probability, ambiguity, complexity, and lack of information as key sources of uncertainty in genetic contexts (Han et al., 2011; Zhong et al., 2020). The perceived effect of uncertainty about how positive results could disrupt major life domains like goals and relationships aligns with previous research on the psychosocial complexities of genetic health threats (Han et al., 2011). Participants' limited genetic literacy, including ambiguity around many gene‐disease links and concerns about testing accuracy, reflects public unfamiliarity with rapidly evolving genomic knowledge. Prioritizing accessible education could improve patient understanding and manage these uncertainties.

Consistent with the UTM, participants exhibited cognitive, emotional, and behavioral responses to the perceived uncertainty. Most participants demonstrated a positive cognitive response, perceiving genetic screening as an opportunity to assess their health status and potentially prevent adverse outcomes. However, our results also highlighted instances where uncertainty led to negative cognitive responses, particularly when considering some gene variations related to the disease that do not have prevention or scientists do not know their effect. Some participants questioned the value of genetic screening in such cases, echoing previous concerns about the limited utility of such information for some patients (Kaphingst et al., 2016). Emotionally, participants reported a range of negative feelings, including stress, fear, anxiety, and worry, consistent with previous literature on the emotional impact of uncertainty in genetic testing (Bottorff et al., 1998; Braithwaite et al., 2002; Rauscher, 2017; Zhong et al., 2021). Interestingly, both positive and negative cognitive responses could elicit negative emotions, suggesting a complex interplay between cognitive and emotional responses to uncertainty.

While previous studies often link uncertainty and negative emotions (e.g., Rafferty et al., 2015; Sahib et al., 2023), there is a lack of research exploring the relationship between uncertainty and positive emotions. The current study observed some positive emotions like calmness, relief, feeling fortunate, and hope. These commonly co‐occurred with cognitive and behavioral responses. It is possible that they act more as outcomes of cognitive processes or behavioral responses to uncertainty, rather than initial reactions to uncertain perceptions. Further studies are needed to explicate the role positive emotions play in the UTM.

Behaviorally, participants exhibited a range of responses, including information‐seeking and avoidance. Information‐seeking was often driven by a desire to alleviate uncertainty and negative emotions, while avoidance was associated with negative cognitive responses and emotions, particularly concerning conditions without prevention or unknown effects. The results were consistent with previous studies conducted in contexts other than genetic screening (e.g. Afifi & Weiner, 2006; Hovick, 2014; Ju et al., 2022).

Aligned with the UTM, the results reveal that cognitive, emotional, and behavioral responses to perceived uncertainty can occur simultaneously and interact with each other. Generally, perceived uncertainty tended to evoke cognitive and emotional responses, which then intertwined and further influenced behavioral responses. While uncertainty perception could directly lead to behavioral responses, this pathway was less common among participants. Notably, positive cognitive and emotional responses do not always translate into positive behavioral responses, as individuals may feel content with their current status and see no need for further action. Similarly, negative cognitive and emotional responses do not necessarily result in negative behavioral responses, as individuals may seek out more information to alleviate their negative appraisals. One possible explanation is that our analysis focused on state UT, while trait UT influences participants' responses (Brown & Fernie, 2015; Eisenberg et al., 2015; Han et al., 2014). Future studies could incorporate individuals' trait UT (Strout et al., 2018) or preference for uncertainty (Ratcliff et al., 2023) information into the UTM to elucidate their relationships with responses to perceived uncertainty.

The study also identified several factors influencing responses to uncertainty, such as family history, religious beliefs, and attitudes toward abortion. Participants unfamiliar with their family history or with a limited understanding of it expressed positive attitudes toward genetic screening, perceiving it as an opportunity to gain insights into their health risks. Conversely, those with a family history of cancer had mixed attitudes, with some viewing genetic screening as unnecessary due to their awareness of preventive behaviors, while others saw value in obtaining additional information. Religious beliefs and attitudes toward abortion emerged as significant factors shaping cognitive and emotional responses to CS. Some participants with strong religious convictions expressed negative attitudes toward CS, stating that the results would not influence their reproductive decisions. Additionally, the reversal of Roe v. Wade influenced participants' views, with some stating that CS would be unnecessary if abortion services were unavailable in their state.

Importantly, the findings suggest potential modifications to the UTM. While the model primarily focuses on uncertainty perception and subsequent responses, our study highlights the role of contextual factors, such as personal beliefs and life experiences, in shaping these responses. Incorporating such moderating factors into the UTM could enhance its explanatory power and applicability across diverse populations and contexts.

This study offers valuable insights for healthcare providers, genetic counselors, and health communicators involved in genetic screening. By understanding the cognitive, emotional, and behavioral responses to uncertainty, healthcare professionals can tailor their communication strategies and support systems to address the specific needs and concerns of women of reproductive age. One key implication is the need for education prior to genetic screening, as most participants demonstrated a lack of knowledge about the connections between gene variations and diseases, and the mechanics and procedures of genetic testing (e.g., ways to get a DNA sample, side effects, test preparation). Participants expressed a desire to receive information, possibly through access to online resources, before their appointments, allowing them to develop a basic understanding of genetic screening and formulate informed questions. Providing accessible and easy‐to‐understand educational materials can empower women to engage in shared decision‐making and alleviate feelings of being overwhelmed during appointments.

Healthcare providers should be prepared to address the uncertainties surrounding genetic screening, particularly those related to gene variations without available treatments or unknown effects. Participants expressed varying levels of interest in receiving such information, highlighting the importance of personalized counseling and respecting individual preferences. Exploring patients' cognitive and emotional responses to uncertainty can guide healthcare providers in tailoring their communication approaches and supporting informed decision‐making.

### 4.1 Limitation

While this study presents valuable insights, it is important to acknowledge its limitations. First, it was conducted at a single site, potentially limiting the transferability of the findings. Additionally, the sample primarily comprised highly educated individuals, most of whom lacked knowledge about genetic screening. This suggests that their perspectives may not significantly differ from those with lower education levels on this specific topic. All interviews were conducted in English, which may have limited participation from individuals with limited English proficiency regardless of their cultural background. This language requirement could have influenced the depth and nuance of responses for some participants and may have excluded perspectives from individuals who would have been more comfortable expressing complex thoughts about genetic screening in their preferred language. Further investigations are warranted to explore how individuals from diverse cultural backgrounds and language proficiencies perceive and respond to uncertainty in genetic screening. Lastly, the qualitative nature of the study precludes establishing causality between uncertainty and participants' cognitive, emotional, and behavioral responses. Therefore, future research endeavors could build on the current study to operationalize the variables in the UTM and integrate longitudinal or experimental designs to elucidate the causal relationships between uncertainty and response patterns and to validate the predispositions proposed by the UTM.

### 4.2 Future research

Several research directions warrant investigation. First, multisite studies with diverse populations, including participants with varying educational backgrounds and limited English proficiency, are needed to enhance the generalizability of findings across cultural and linguistic contexts. Second, longitudinal and experimental designs should be employed to establish causal relationships between uncertainty perceptions and responses, while developing validated quantitative measures of UTM variables to enable larger‐scale testing. Third, future research should examine how individual difference factors, such as trait UT and cultural beliefs, moderate responses to genetic screening uncertainty. Finally, intervention studies are needed to develop and test educational and communication strategies that effectively manage uncertainty while supporting informed decision‐making in diverse populations. These investigations would advance both the theoretical understanding of the UTM and practical applications in genetic counseling practice.

## 5 CONCLUSION

The current study contributes to our understanding of how women of reproductive age perceive and respond to uncertainty in the context of genetic screening. By drawing upon the UTM, the findings highlight the cognitive, emotional, and behavioral responses to uncertainty, as well as the factors shaping these responses. While most participants demonstrated interest in genetic screening, perceiving it as an opportunity for health assessment and prevention, uncertainties surrounding gene variations without available treatments or unknown effects elicited negative cognitive and emotional responses. The study underscores the importance of comprehensive education and tailored communication strategies to address the specific needs and concerns of women navigating uncertainty in genetic screening. Healthcare providers, genetic counselors, and health communicators can leverage these findings to develop resources and support systems that promote informed decision‐making and manage uncertainty effectively.

## AUTHOR CONTRIBUTIONS

**Yi Liao:** Conceptualization; formal analysis; methodology; writing – original draft. **Anne C. Madeo:** Methodology; writing – review and editing. **Lingzi Zhong:** Writing – review and editing. **Wendy Kohlmann:** Writing – review and editing. **Erin Rothwell:** Writing – review and editing. **Kimberly A. Kaphingst:** Conceptualization; funding acquisition; supervision; writing – review and editing.

## CONFLICT OF INTEREST STATEMENT

The Authors have no conflict of interest to report.

## Data Availability

The interview transcripts analyzed during the current study are not publicly available due to privacy concerns but are available from the corresponding author upon reasonable request.

## APPENDIX A INTERVIEW GUIDE

### A.1 INTRODUCTION

During this interview, we will be talking about information that people could learn from having genetic testing. This means a laboratory looks at a person's genes to be able to see what variations they carry in their genes. A variation means that the version of the gene that a person carries is different from the version carried by most other people.

People have many different types of gene variations. Some variations could affect the risk of cancers or other diseases that could be prevented or screened for; some might affect the chance of getting diseases that cannot be prevented or treated. Some variations might not affect a person's own health but could cause serious health issues in children if both parents carry the variation, and, in many cases, the effect of a variation is unknown. It is important to know that in many cases, having a gene variation does not mean that you will certainly develop a condition.

**I'd first like to ask you some general questions about what you might be interested in learning about your genetic information**.

1) If you were able to find out information about the gene variations that you might carry, what would you hope to learn?

***Probes***

- Some gene variations mean that you have an increased risk for diseases or health conditions where there are steps you can take to prevent or treat the disease. Would you want to find out this type of information? Why or why not?
- Would you want to find out if you were at increased risk for disease or health conditions for which there is no treatment or prevention? Why or why not?
- Would you want to find out about variations where we do not yet know the effect on health? Why or why not?

**OK, I'd next like to talk about two specific types of information that you could receive from genetic testing. I'll describe each possible type of information to you, and then ask you some questions about your thoughts on that type of information**.

**One type of information that you could learn from genetic testing is about a variation in one of your genes that increases your risk of developing a cancer that could be prevented or treated. For example, you could learn that you have a gene variation that means that you are more likely to develop colon cancer**.

2) How interested would you be in genetic testing that could tell you more about your risk of developing cancer? Why is that?

*[Note: If the participant respondents that it depends upon insurance coverage, you can say “What if the genetic testing was fully covered by your insurance?”]*

***Probes***

- What might make you more interested in this type of information?
- What might make you less interested?
- What meaning would this information have for you personally?

3) What types of cancers would you be most interested in finding out your risks for? Why is that?

4) Are there any cancers that you would not want to find out your risks for?

***Probes***

Which cancers? Why is that?

*[Note: For Questions 3 and 4, if the participant asks what others have said or for more prompting, you can say “We are most interested in what comes to mind for you.”]*

5) In thinking about getting genetic test results that would tell you about your risk of cancer, how important is it to you that prevention or treatment steps are available? Why is that?

***Probes***

- For example, for breast or colon cancer, you may be able to have more frequent or earlier screenings to help find cancer at an earlier stage. For other types of cancer, we do not have prevention or treatment steps available.

6) How interested would you be in receiving this type of genetic testing as part of a routine appointment with your gynecologist? Why is that?

6b) What would you want to be told about this type of genetic testing from your gynecologist?

7) How interested would you be in using an automated computer education program to learn about this type of testing?

8) How interested would you be in receiving this type of genetic testing in the next year? Why?

**Another type of information that you might learn from genome sequencing is about a variation in one of your genes that does not affect your health, but could cause health problems if passed on to a child. For example, you could learn that you have a variation in the gene that causes cystic fibrosis or the gene that causes sickle cell disease. Having a variation in this type of gene would not cause any health problems for you. But if you were to have children with someone who also had a variation in the same gene, then your children could be born with these serious diseases**.

9) How interested would you be in learning this type of information about a gene variation that does not affect your health but might affect the health of your children? Why is that?

*[Note: If the participant respondents that it depends upon insurance coverage, you can say “What if the genetic testing was fully covered by your insurance?”]*

***Probes***

- What might make you more interested in this type of information?
- What might make you less interested?
- What meaning would this information have for you personally?

10) Would your interest in this type of genetic testing depend on whether you were actively planning to become pregnant? Why is that?

***Probes***

- If you were planning to become pregnant, do you think you would want this information before pregnancy? Why or why not?
- Would you want this information during pregnancy? Why or why not?

10b) [If a participant has mentioned that they have biological children] Do you wish you had access to genetic testing before your first pregnancy? Why or why not?

- What would that information have meant for you?

11) How interested would you be in receiving this type of genetic testing as part of a routine appointment with your gynecologist? Why is that?

11b) What would you want to be told about this type of genetic testing from your gynecologist?

12) How interested would you be in using an automated computer education program to learn about this type of testing?

14) How interested would you be in receiving this type of genetic testing in the next year? Why?

15) How would you compare your interest in learning this information to your interest in learning about your risk of cancer? Why is that?

16) Thinking about both types of genetic test results that we have discussed, how interested would you be in being tested for both at the same time? Why is that?

- Would you be interested in receiving additional genetic tests at that time? Why or why not?

*[Note: If the participant asks what type of additional genetic tests, you can say “We are most interested in what comes to mind for you. Is there any genetic information that you would be particularly interested in learning?”]*

17) Thinking about both types of genetic test results that we have discussed, how might this information impact your life if you did have genetic testing?

- Who would you talk to about this information? Why?
  ◦ Your gynecologist? Another health care provider?
  ◦ Your partner or other family members?

**My last questions are to find out more about you**.

15) What is the highest level of schooling that you have completed?

▢ Elementary school
▢ Junior high or some high school
▢ High school diploma or Graduate Equivalency Diploma (GED)
▢ Some college or Associate degree
▢ College degree or higher

16) How would you describe your race and ethnicity? You can choose multiple categories.

▢ White/Caucasian
▢ Black/African American
▢ Hispanic/Latino/Latinx
▢ Pacific Islander or Native Hawaiian
▢ Asian
▢ Other group

17) Have you had biological children?

▢ Yes
▢ No

18) If yes to Q17: Did you have any type of genetic testing with your prior pregnancy(ies)?

▢ Yes
▢ No
  If yes: Do you remember what the tests were for? ____________________
▢ Not sure

19) Are you actively planning to become pregnant in the next year?

▢ Yes
▢ No
▢ Not sure

**That is the end of the questions that I'd like to ask you. Do you have anything else that you would like to tell me?**

**Thank you again for agreeing to share this information with us**.

## APPENDIX B CODEBOOK

**The perception of uncertainty:** The conscious, metacognitive awareness of ignorance, which may stem from a lack of knowledge about accessing genetic testing, understanding how genetic testing works, etc.

**Example**

“I don't know which cancers all have been linked genetically.”

“But I mean like for genes, like I don't really care about things like eye color, you know, different things like that. I don't even know if you can like test that with genes, but like‐‐ because I don't know that much about genes.”

“I've never had someone like talk to me about genetic testing like at a doctor's in regard‐‐ well, except for like in regards to like whether that's something I was interested in for the baby.”

“I think sometimes like **unknown** creates a little bit of anxiety, but I really believe that knowledge is power.”

### B.1. Cognitive response

#### B.1.1. Positive

- Opportunity: Genetic testing represents a chance for individuals to gain insights into their cancer risk (carrier risk), enabling them to take preventive measures earlier.
- Acknowledgment: Recognition of the value and significance of genetic testing in managing health risks.
- Confidence: After processing information and considering the implications, individuals form a level of confidence in their decision to pursue genetic testing or in the actions they choose to take based on the results.
- Show interest and curiosity about genetic testing.

**Example**

“I've always felt that as long as someone is like more informed, it's just, I think, better.”

“I would like to get everything or try to be as healthy as I can be, know any illnesses or anything.”

“I think if we were having problems I would want to know if I was a carrier for something that my husband was also a carrier for because I think that would determine how we would go about conceiving kids.”

“I guess if it was more offered to me like at my annual physical or something then I'd be more apt to do it than‐‐ I don't think it's something that I think about enough that I would go out and search to get screened if that makes sense.”

“Just curious‐wise, I guess. I know medical is still going a long way. **So, maybe there is in the future and just knowing now, maybe I can catch my eye on it later**.”

“Just **curious‐wise**, I guess. I know medical is still going a long way. So, maybe there is in the future and just knowing now, maybe I can catch my eye on it later.”

“I think, you know, obviously you said that we would take out like the cost factor, you know, if it was covered by insurance that would be something I'd be more interested in it.”

#### B.1.2. Negative

Threat; denial; vulnerability; doubt; disinterest.

- Question the value of genetic testing.
- Concern about the side effect of getting test or the impact of test results.
- Lack of self‐efficacy.

**Example**

“Cost is a big issue there.”

“I would be **a little less interested** just because I'm an anxious person and I don't know if I want to add one more thing to worry about. But I think I would still do it if it was offered.”

“**Less interested than cancer screening**, but if it was offered to me and I knew my insurance covered it, I would probably still do it.”

“I think if something could or couldn't happen in the future, sometimes it's better‐‐ **I think it could negatively impact your life in the present because you'd be thinking about that possibility too much when you don't know**. We don't really know and so, I think it could negatively impact your current state of mind if we would‐‐ that's probably more me. I just know that “You know what? I'm just going to…”.”

### B.2. Emotional response

#### B.2.1. Positive

- Hope: Feel hopeful about the potential outcomes of genetic testing. This hope can be related to the possibility of taking proactive steps toward managing their health, preventing disease, or preparing for the future based on the knowledge gained from the test results.
- Relief: If they have been living with uncertainty about their risk for a genetic condition, the prospect of gaining clarity and answers can be comforting.
- Confidant/calm: Feel content about the current situation or their decision; may relate to religious belief.

**Example**

“I'm **not too worried** based on what I know about our families, but there's stuff in our family genetics we don't know as much about.”

“I actually don't really know what our reasoning was. We just **weren't that concerned** and so we didn't do genetic testing.”

“Mm, I think for that question, I wouldn't be interested. The only reason I did the test for baby is because of the gender. <laughs> I didn't really‐‐ if the baby has something, some genetic abnormality then that's what it was‐‐ **that's how it was meant to be. Like that's how I guess God wanted it**.”

#### B.2.2. Negative

Worry; fear; despair; surprise; anxious (including personal trait).

**Example**

“I would like to like know anything in advance just so **I don't get any surprises** maybe like later or something.”

“It's just I've always been kind of just **scared of** not finding out like any like diseases or illnesses that I might not know of that could maybe be in me. I've been really always really kind of like **scared of** diseases that I don't know and not being able to treat it on time. Yeah.”

“I've always been kind of just **scared** of not knowing what I might have.”

“No, I think I'd be too **anxious** about it.”

“I would be a little less interested just because I'm an **anxious person** and I don't know if I want to add one more thing to **worry about**. But I think I would still do it if it was offered.”

“I think sometimes like unknown creates a little bit of anxiety……”

### B.3. Behavioral response

#### B.3.1. Positive

Approach; action; decision‐making; info seeking. (Sometimes they will mention what they want to find out).

**Example**

“I would hope that I'd learn what could affect me in the future and what I could possibly pass on to my kids.”

“Just what exactly they're screening for because I mean it's not all encompassing, right, they look for very specific markers. So I would just want to know what markers they're looking for.”

“I think it would be important for her to describe what tests they were doing, how confident they are in the tests, how much the effectiveness of being able to predict different things and then also, describing the pros to getting it done, also, the risks and for sure, describing like what would happen based on the results, what the next steps would be based on the results.”

#### B.3.2. Negative

Avoidance; inaction; decision deferral; inattention.

**Example**

“This kind of testing, I think that I‐‐ I know it's bad, but I just wouldn't‐‐ I don't think I would want it at all.”

“**No**, I think I'd be too anxious about it.”

“Unless I would have had any like really specific concern, **I wouldn't do that during pregnancy**.”
